# Protecting cell cycle integrity: enhanced start-codon stringency in mitosis

**DOI:** 10.1038/s41392-024-02123-5

**Published:** 2025-02-05

**Authors:** Omid Omrani, Kanstantsin Siniuk, Martin Fischer

**Affiliations:** Computational Biology Group, Leibniz Institute on Aging – Fritz Lipmann Institute (FLI), Jena, Germany

**Keywords:** Cell biology, Molecular biology

A recent study published in *Nature* has uncovered a novel regulatory mechanism that enhances start-codon selection during mitosis in mammalian cells by intensifying the interaction between the 40S ribosome subunit, which binds messenger RNA (mRNA) and initiates translation, and the eukaryotic translation initiation factor 1 (eIF1), a central regulator of start-codon selection.^1^ This discovery reveals a sophisticated layer of translational control that helps maintain cell viability and cell cycle stability, with potential implications for understanding cellular regulation and improving cancer therapies.

The cell cycle is a tightly regulated process through which cells grow and duplicate. It consists of four main phases: gap 1 (G1; growth), DNA synthesis (S), gap 2 (G2; preparation), and mitosis (M). A complex system monitors internal and external conditions, ensuring appropriate and timely progression to the next phase. These regulatory layers prevent errors that could cause cellular instability or uncontrolled growth, which are hallmarks of diseases like cancer. Mitosis is particularly critical because errors during chromosome segregation can lead to cell death or contribute to disease.

Cell cycle regulation involves multiple layers, including phosphorylation by cyclin-dependent kinases (CDKs), degradation via the ubiquitin system, and transcription regulation. CDKs, activated by growth signals, phosphorylate target proteins to drive cell cycle progression. The ubiquitin system selectively degrades specific proteins at critical transition points, while transcriptional regulators, including the E2F family, the retinoblastoma protein family, B-MYB, and FOXM1, create waves of gene expression that shape the G1/S and G2/M phase transitions.^2^ Together, these interconnected layers ensure the cell cycle proceeds with precision.

Translational regulation—controlling which proteins are synthesized from existing mRNAs—is another crucial control layer, especially during mitosis, when transcription is reduced. Typically, mRNAs contain a 7-methylguanosine (m^7^G) cap at their 5’ end, which is recognized by cap-binding proteins that recruit the 40S ribosome subunit along with cofactors.^3^ This complex scans the mRNA for a start-codon, typically AUG, although alternative codons such as CUG, AUU, and GUG, among other closely related codons, can also mark the beginning of translation.^4^ The presence of a Kozak sequence, a specific nucleotide pattern flanking that start-codon, enhances recognition efficiency. Once identified, the 60S ribosomal subunit joins to form the 80S ribosome that translates the mRNA into protein. Importantly, cells can use alternative start-codons to produce protein variants that contribute to functional diversity. Key cofactors like eIF1 and eIF5 regulate the stringency of start-codon selection, balancing canonical and alternative isoforms.^3,4^

Ly et al. investigated the role of translation during mitotic arrest using S-trityl-l-cysteine, a spindle motor protein inhibitor. They observed that mitotically arrested cells are highly sensitive to translation inhibition, indicating that active protein synthesis is essential for cell viability during prolonged mitosis.^1^ The researchers employed ribosome profiling, a technique that maps ribosome-bound RNA fragments, and RNA sequencing to infer translation initiation sites in both mitotically arrested cells and non-mitotic (interphase) cells. Their analysis revealed that during mitotic arrest, cells suppress the production of alternative translational isoforms by prioritizing the canonical AUG start-codon flanked by strong Kozak sequences.

**Fig. 1:**
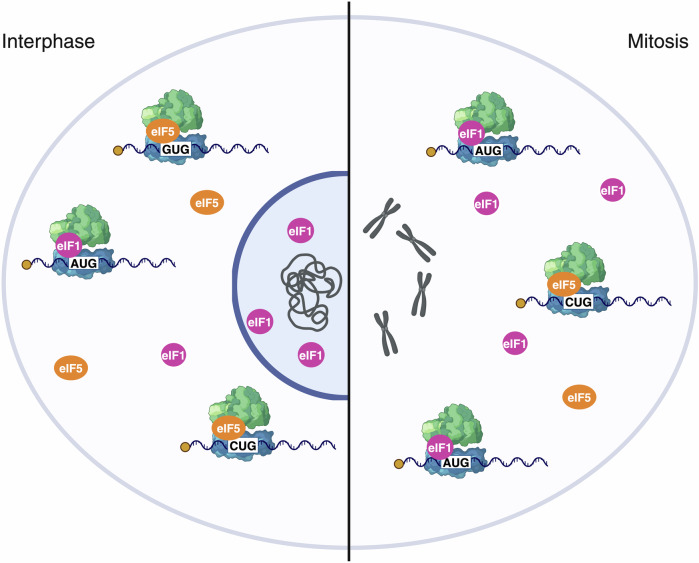
A nuclear reservoir of eIF1 enhances translation initiation stringency during mitosis. In interphase, eIF1 is sequestered in the nucleus, limiting its availability for ribosomal association. This sequestering allows more frequent interactions between ribosomes and eIF5, which facilitates translation initiation at alternative start-codons. Upon nuclear envelope breakdown during mitosis, eIF1 is released into the cytoplasm, where it associates with ribosomes, increasing the proportion of ribosomes that initiate translation at the canonical AUG start-codon, thereby enhancing translation initiation stringency. The figure was created in BioRender. Omrani, O. (2025) https://BioRender.com/f64o283

To understand the mechanisms, the researchers assessed 40S subunit protein interactions using sucrose gradient fractionation followed by mass spectrometry. They identified increased eIF1 binding and decreased eIF5 interaction with 40S during mitosis,^1^ consistent with their roles in regulating start-codon recognition stringency. Further experiments showed that a large fraction of eIF1 was located in the nucleus during interphase and released to the cytoplasm when the nuclear envelope breaks down in mitosis, acting as a regulatory switch as the cell cycle progresses into mitosis (Fig. 1).

The authors also demonstrated that cells lacking the nuclear reservoir of eIF1 were more prone to cell death during mitotic arrest but less likely to exit mitosis prematurely.^1^ While many protein isoforms with small N-terminal deletions or additions are likely to function similarly to the canonical isoform, some may have altered functions. For instance, previous findings from the same group revealed that alternative translation initiation affects CDC20, a key spindle assembly checkpoint protein. Shorter CDC20 isoforms, produced by leaky ribosome scanning past the first AUG, are less effective in delaying mitotic exit under stress.^5^ In their current study, the Cheeseman group shows that the increased association of eIF1 with 40S suppressed the first AUG, leading to higher translation of the shorter, less functional CDC20 isoform, which contributes to premature mitotic exit.^1^ This pathway appears to balance between cell death and premature exit from mitosis when stress leads to a delay in mitosis.

An additional observation by Ly et al. was the preference of the eIF1:40S complex for translating nuclear-encoded mitochondrial mRNAs during mitosis.^1^ This suggests that translational control may be particularly important for the fitness of mitochondria during prolonged mitosis, a phase characterized by high energy demands.

These findings have potential implications for cancer therapy, particularly for drugs targeting mitotic arrest, such as taxanes and vinca alkaloids. While these substances induce cancer cell death during mitotic arrest, some cells escape through premature mitotic exit (also known as mitosis slippage), potentially allowing cells to continue proliferation and obtain new properties due to chromosome segregation errors. Targeting translational initiation factors such as eIF1 could offer new strategies to manipulate cell fate under these conditions.

Beyond cancer, this discovery may also have relevance in contexts like oocyte maturation, where transcription is limited for extended periods. Future research will be crucial to determine the broader applicability of this mechanism and explore its potential therapeutic relevance. Overall, these findings highlight translational regulation as a critical layer of cell cycle control.
